# Biomechanical Characteristics of the Typically Developing Toddler Gait: A Narrative Review

**DOI:** 10.3390/children9030406

**Published:** 2022-03-13

**Authors:** Wei Liu, Qichang Mei, Peimin Yu, Zixiang Gao, Qiuli Hu, Gustav Fekete, Bíró István, Yaodong Gu

**Affiliations:** 1 Faculty of Sports Science, Ningbo University, Ningbo 315211, China; liuwei2@nbu.edu.cn (W.L.); pyu926@aucklanduni.ac.nz (P.Y.); gaozixiang0111@outlook.com (Z.G.); huqiuli@nbu.edu.cn (Q.H.); 2 Research Academy of Grand Health, Ningbo University, Ningbo 315211, China; 3 Faculty of Engineering, University of Pannonia, 8200 Veszprém, Hungary; fg@inf.elte.hu; 4 Auckland Bioengineering Institute, The University of Auckland, Auckland 1010, New Zealand; 5 Faculty of Engineering, University of Szeged, 6724 Szeged, Hungary; biro-i@mk.u-szeged.hu

**Keywords:** toddler, foot, biomechanics, plantar pressure, gait development

## Abstract

**Simple Summary:**

This narrative review clarified the gait biomechanics of typically developing toddlers and revealed the changes of gait characteristics at different age stages till independent walking. The remarkable gait characteristics and developmental nature of toddlers indicate that the gait pattern of the junior independent walkers differs from the senior and experienced cohort. Gait patterns are associated with neuromuscular maturation. Changes in gait biomechanics are age-dependent. Therefore, it is necessary for pediatric clinicians to understand the characteristics and stages of normal or abnormal development. Developmental neuromotor control suggests that early identification and intervention may expedite treatment and optimize outcomes.

**Abstract:**

Independent ambulation is one of the most important motor skills in typically developing toddlers. Gait analysis is a key evaluation method in basic and clinical research. A narrative review on the literature of toddler gait development was conducted following inclusion criteria, explicitly including the factors of English article, age range, no external intervention during the experimental process of studies involved, the non-symptomatic toddler, and no pathological gait. Studies about toddlers’ morphological, physiological, and biomechanical aspects at this developmental stage were identified. Remarkable gait characteristics and specific development rules of toddlers at different ages were reported. Changes in gait biomechanics are age and walking experience-dependent. Gait patterns are related to the maturation of the neuro and musculoskeletal systems. This review thus provides critical and theoretical information and the nature of toddler walking development for clinicians and other scientific researchers. Future studies may systematically recruit subjects with more explicit criteria with larger samples for longitudinal studies. A particular design could be conducted to analyze empirically before practical application. Additionally, the influence of external interventions on the development of toddler gait may need consideration for gait development in the toddler cohort.

## 1. Introduction

Human gait is a motion state of the motor system during walking, which is the process of moving in a particular direction with a series of continuous and coordinative activities of hip, knee, foot, and ankle. In other words, gait refers to a movement pattern of limbs, especially lower limbs on a substrate, which can fulfill the primary need of locomotion and provide propulsion and support for the body [1]. Behind the gait lies several key indications of neuromusculoskeletal growth and development. Toddlers are in a critical period of independent walking. Therefore, a systematic and comprehensive understanding of toddlers’ gait is important. During this stage, toddlers experienced dynamic and progressive changes, such as rapid growth and development of anatomical, neuromuscular, and sensory systems [2], the ossification of bones [3,4], and the appearance of the foot arch structure [4]. Children are considered infants, if less than or equal to 12 months, as toddlers between 13 and 36 months [5]. 

Clinical examination and locomotor milestones are the mainstream means to identify potential motor impairment in toddlers. However, such assessments present non-specific characteristics, which challenge the evaluation of locomotor milestones. For example, regarding the onset of independent walking, it is reported that this process occurs at the age of 12 months, while 10% of the typically developing toddlers cannot walk independently until 14.4 months or later [6]. Not all alternative gaits are caused by diseases, toddlers also exhibit this feature during walk learning. The finding indicates that toddler gait is influenced by a variety of factors, such as age, walking experience [7], body dimensions [7,8,9], maturation of central nervous system [9,10], the muscle-fat ratio [11], development of musculoskeletal system [12,13], and head-trunk posture stability [14].

The research of gait has been relatively mature up till now. With the development and improvement of measurement technologies, researchers can carry out dynamic quantitative analyses on the characteristics of human locomotion. It is vital to understand or master the gait differences between typically developing toddlers and adults to describe, study and treat abnormal gaits in toddlers. Several cross-sectional and few longitudinal studies have analyzed toddler gait at the onset of independent ambulation, including spatiotemporal characteristics [15,16,17,18,19,20,21,22,23,24,25], plantar pressure, and kinematics [8,25,26,27,28,29,30,31,32,33,34]. Predecessors have done similar studies. For example, Price et al. summarized existing literature quantifying biomechanical characteristics in toddler cruising, supported, and independent walking [35]. The current review is more focused on the independent walking of toddlers since 12–36 months, which is a developmental stage after the transition from sitting to walking.

To describe, study and take immediate treatment of abnormal gait, researchers should master the differences between toddler and adult gait, the time when toddler gait is mature, and the factors influencing gait maturation [12]. Further research, especially more longitudinal studies, may investigate biomechanical characteristics of the typically developing toddler gait. The present review aims to clarify and reveal the changes of gait characteristics of toddlers at different age stages to independent walking. The remarkable gait characteristics (or parameters analyzed), such as plantar pressures, joint motion, moment, and specific developmental nature of toddlers are reported, guiding clinicians, scientific researchers, and even parents in gait evaluation, correction, and development.

## 2. Method

While collating and reviewing relevant literature, the authors found that the diversity and quantity of literature related to gait development in the typically developing toddlers were sufficient. However, due to individual differences in the growth and development of toddlers, there is no unified age range of toddlers. Meanwhile, the authors aimed to summarize and critique literature, so we chose a narrative literature review rather than a systematic review for this study.

### 2.1. Information Sources

A comprehensive and reproducible search strategy was performed in the following databases (PubMed, Web of Science, and Google Scholar) from January 2000 to December 2021.

### 2.2. Search Strategy

The search terms used in each database are as follows, (1) In PubMed, the search string is “(((infant*) OR (child*) OR (toddler*)) AND (gait)) [Title/Abstract].” (2) In Google Scholar, the search string is “(((infant) OR (child) OR (children) OR (toddler gait)) AND (gait)) [Title].” (3) In Web of Science, the search string is “(((infant*) OR (child*) OR (toddler*)) AND ((gait) OR (gait development) OR (developing gait))) [Title].”

### 2.3. Inclusion and Exclusion Criteria

Reference lists from identified literature were manually searched for completeness by the authors to confirm content relevant to the development of the toddler gait. Studies were included for this review if met the following eligibility criteria: (1) written in English, (2) age range (studies with subjects between 10–36 months were included), (3) no external intervention during the experiment of the study involved (For example, the effects of clothes, shoes and visual environmental distraction), (4) no pathological gait, (5) the non-symptomatic toddler.

Studies were excluded if met the following criteria: (1) citations and patents, (2) review articles, (3) written in non-English, (4) abstract papers without data, (5) other no considered gait characteristics (For example, muscle activation and energy expenditure were excluded).

### 2.4. Risk of Bias in Individual Studies

The Cochrane Collaboration Risk of Bias Assessment Tool was used to evaluate the risk of bias in individual studies. Two independent authors (Wei Liu and Qichang Mei) evaluated all the included studies, and any disagreements were discussed. An independent arbitrator (Bíró István) was invited when an agreement was not met.

## 3. Results

**Figure 1:**
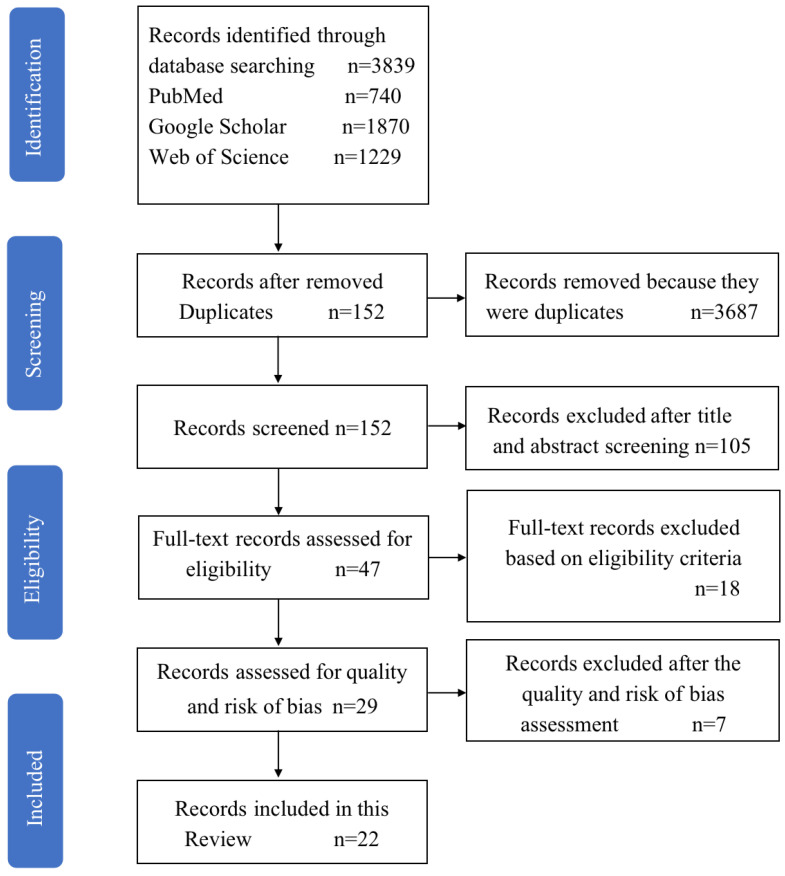
The flow chart of the literature inclusion in this review.

The search yielded 3839 titles and abstracts for initial screening. A total of 47 full texts were screened, and 25 were excluded. Twenty-two studies were included for the final analysis. The identification process was illustrated by a flow chart in Figure 1. Based on the information of all the full texts included, the results of data collection and summary measurement of each included study were presented in Table 1 and Table 2. The details included authors, study site, participants, age, design, measurement frequency and timing, data collected, anatomic sites, walking speed and style, footwear/attire, instruments, variables.

## 4. Discussion

### 4.1. Spatio-Temporal Characteristics of Typically Developing Toddler Gait

Toddlers develop rapidly in structure and function as growth. Expression of alternative gait patterns might require neuromuscular maturation and learning time during the period of independent walking [36]. As an essential supporting structure, the toddlers’ foot has experienced dynamic and gradual changes, such as the bone and arch structures [4]. These alterations led to apparent differences in toddlers’ locomotion strategies. Changes in gait biomechanics were age-dependent, and the gait parameters, such as stride length, step width, and duration of swing, vary with age [12,17,20,25,37,38,39,40,41]. The changes in these parameters were closely related to balance, coordination, and metabolic cost [3,5,6,7,9,16,42,43,44,45,46,47].

#### 4.1.1. Temporal Parameters of the Toddler Gait

While walking independently for about two months, the temporal parameters showed developmental changes, i.e., stride time, stance time (stance time as a percentage of stride time), cadence, and normalized cadence. These parameters presented a turning point two months after toddlers walked independently (decreasing from the first week of independent walking (T0) to two-month after independent walking (T2) and increasing from T2 to six-month after independent walking (T6)) [16]. Time gait cycle parameters increased with age, while cadence decreased [12,37,38]. At the same time, swing time as a percentage of stride time increased from a value of 40–50% at T0–T2 and then remained constant [16]. Novice and experienced walkers spent 42.5% and 33.9% of the stance phase in the double support [22]. This finding was slightly lower than the result obtained by Clark et al. that double support time of junior independent walkers was from 20% (adult referral value) to 60% [47]. The study [22] observed toddlers aged 7–13 months in the non-laboratory environment (Inclusion criteria for the novice group, hands in high-guard position, uncertainty during walking, a lack of stability and control), and it could be the reason why the results were lower. The temporal characteristics of immature gait patterns were a broad base of support, prolonged stance duration [12,39], and increased double support [12,25,39]. The changing trend of the above parameters showed a gradual decline close to the adult standard, suggesting the transition process of toddler gait becoming gradually matured [25].

As the walking experience increased, toddlers acquired the balance strategy. During this stage, the walking cadence and speed increased [15], but faster-walking speed was due to the high cadence rather than step length [21]. However, Bisi et al. [16] and Bril et al. [48] considered that increase in walking speed was due to the long step length rather than cadence. Sutherland et al. thought that increased leg length without an increase in cadence would increase the walking speed with a stable walking pattern [12]. The differences in the above perspectives might lie in the results between different sample sizes and ages. A more extensive study might better understand the relationship between step cadence and speed in early walking development [21]. A large-scale biomechanical gait parameters database of healthy junior children (including toddlers aged 1 to 3) have been built by Hamme et al. The databased presented an original regression of parameters with age, walking speed, and the age–speed interaction and deduced the typical reference targets from regressions [24].In any case, the growth (faster and longer) of steps was a sign of a more mature gait in toddlers [17,19,49].

Different views on the relationship between gait cadence, age, and walking experience were reported, for example. Gait cadence increased significantly with age and walking experience [17,21,22]. Owen et al. denoted that cadence showed a slight downward trend with the increase of age [12,37,38]. Guffey et al. showed that standard cadence decreased by 15% from 2 to 4 years [20]. However, Bisi and Stagni presented that cadence increased first and decreased from novice walkers to stable walkers [16]. Current studies agreed that the changing nature of gait cadence could be mainly divided into several stages. At the beginning of walking (3–6 months after the onset of independent walking), toddlers concentrated on overcoming gravity to maintain postural gait requirements, gradually to the fine-tune gait patterns [16,17,21,49]. In general, a relatively high gait cadence was observed at the beginning of walking. When toddlers slowly grasped the skill of gravity, the gait cadence decreased gradually and eventually remained stable after adulthood [22].

#### 4.1.2. Spatial Parameters of the Toddler Gait

At the initiation of toddlers’ independent walking, a toddler strategy was employed. According to the findings of Bisi and Stagni, toddlers have determined the different gait strategies in the first month of independent walking [16]. Whitall and Getchell believed that individual walking and running techniques appeared after 9.5 months of independent walking [50]. The spatiotemporal characteristics of gait strategies changed the loading mode of feet, mainly manifested in the increase of step frequency (or cadence) to keep balance [21,23,51]. At the onset of independent walking, toddlers who took relatively wider steps, longer steps, and a shorter swing duration had a large normalized spatial stability margin. However, with the increase of age, the spatial margin of stability would gradually decrease [18]. The mid-lateral distance was significant [15], and step width exceeded step length. Over the next few months, toddler walking improved significantly, and step width diminished while walking speed and step length increased gradually [12,15,17,21,23,51]. Smaller step width provided stability and narrowed as the balance improved [25,48,52,53,54], which showed the trend of mature gaits close to adulthood [51].

In addition, step and stride length increased with age, and there were significant differences between step and stride length and age groups [20]. Toddlers walked slowly between 10 and 15 months, with steps shorter than leg length [17,40]. A few months later, speed and step length dramatically increased [17], and occasionally, steps larger than leg length could be observed [40]. Dusing and Thorpe have also proved that normalized step length and velocity increased from 1 to 4 years old [37]. However, Moe-Nilssen et al. measured the movement of lower limbs through a triaxial accelerometer and demonstrated that stride regularity and step regularity had a low frequency in the toddler gait [55]. Bisi et al. also found no significant increase in the regularity [16]. Perhaps a more comprehensive longitudinal study should focus on how these parameters would change during gait maturation in the future. The data obtained by Guffey et al. also demonstrated that the difference of normalized spatial-temporal parameters in different age groups was not statistically significant [20]. Other normalization methods might cause the difference mentioned above. Dusing et al. utilized the height [37], and Guffey et al. believed that leg length was more suitable for normalizing the spatial dimensions [20], causing the proportion between the leg and torso of toddlers to change gradually. With the continuous maturity of gait, toddlers’ independent walking stability increased continuously. However, normalized gait parameters were mature after three years old [18].

To adapt to the maturation of locomotor patterns, i.e., walking and running, toddlers needed to constantly alter the interlimb coordination [42,43,44,45,46,56,57]. Hallemans et al. suggested an early gait maturation after four months of independent walking [25]. Bril and Breniere confirmed that toddlers showed developmental changes with 5–6 months of independent walking experience [48]. Van Dam et al. indicated a relation between morphology (the head and pelvis) and the step-time parameters of gait in toddlers between 15 and 36 months [19]. Unlike standard laboratory environments, non-laboratory investigations (an open-sourced toddler gait video analysis) also described how toddlers walk in familiar settings. With the increase of walking experience, the frequency of toddler falling gradually decreased [22]. A study compared the traditional straight-path task with spontaneous walking in 97 toddler gait characteristics, which correlated highly with each other. The free-play task benefited understanding improvements in walking to control balance and forces. Meanwhile, gait characteristics during spontaneous walking had implications for studying the development of walking in toddlers with impairments [15].

### 4.2. Kinematic Characteristics of Typically Developing Toddler Gait

Special attention was paid to the foot kinematics while collating and reviewing literature related to the development of gait in toddlers. Whether in mechanical energy or kinematics, the ability to walk slowly developed from initial independent steps to about seven years old [33,34,49,58,59]. Toddlers needed to constantly change the interlimb coordination, which was a slow development process [36]. The gait of toddlers aged 10–15 months was characterized by high variability and low stability [60]. Walking independently for about one month, toddlers started to show the characteristics of the pendulum mechanism [16,33].

Zeininger et al. reported that the initial heel contact steppers went through lower vertical forces at impact, indicating the absorption of peak force by knee yielding or the transition to the heel-toe contact pattern [8]. Knee yielding followed heel strike [17], similar to adults [61], further illustrated that the initial heel contact mode was the transition to adult gait. Flat foot contact steppers bore higher vertical forces and appeared the rapid downward trajectory of the foot and leg, showing a less yielding gait [8]. A conclusion was supported by Hallemans et al., suggesting that the knee did not yield or absorb energy between two weeks and five months of walking experience for toddlers [25]. There were no significant differences in ankle angle between the initial heel contact and flat foot contact [8].

In addition, age had a significant influence during the pre-swing phase, according to the study of Samson et al. [31]. With an increase in age, knee flexion decreased [26]. The maximum flexion of the metatarsophalangeal joint increased with age. However, there was no significant difference between different age groups, indicating that metatarsophalangeal joints were more passive and matured faster. There was also no significant difference in dorsiflexion/plantarflexion and inversion/eversion between different groups [31]. Hallemans et al. observed slight plantar flexion movement of the ankle was likely to be a passive movement, possibly coming from gravity on foot segment in toddlers (aged 13.5 to 18.5 months) [25]. Another follow-up longitudinal study by Hallemans et al. observed that ankle plantarflexion at foot contact and maximal hip extension in stance increased with increasing walking experience [17]. By comparing kinematics and EMGs in toddlers (aged 12 to 15 months) at the onset of independent walking with or without hand or trunk support, Ivanenko et al. [34] indicated that immaturity of global gait parameters did not depend on postural stability. Even with or without support, toddlers still exhibited a characteristic gait pattern until the occurrence of the first unsupported steps and rapidly matured thereafter.

### 4.3. Kinetic Characteristics of Typically Developing Toddler Gait

#### 4.3.1. Ground Reaction Force Related Parameters of the Toddler Gait

Previous studies reported that toddlers transited from the early flat foot contact to the initial heel contact and then developed towards adult gait patterns [17,62,63,64,65,66]. This alteration was a typical mature contact pattern in the process of human gait. Consistent initial heel contact usually occurred at 12 months after independent walking [39]. This view was also supported by Sutherland et al. [12] and Hu et al. [27], suggesting that the heel-strike model did not appear until two years old.

Under a laboratory setting, the ground reaction force of toddler gait during walking was examined by analyzing the kinetic data of 18 toddlers (aged 11.5–43.1 months) [8]. The initial heel contact and flat foot contact steps differed significantly in the location of central pressure relative to the calcaneus. Toddlers with flat feet at touchdown showed the characteristic of wide heel in the morphology of feet and less walking experience. The morphological changes would assist in reducing the heel loading at the onset of toddler independent walking and frequently shift the center of pressure in front of the heel [8,67,68], showing an evenly distributed plantar loading. Compared with the pattern of flat foot contact, the toddlers who landed with initial heel contact were more dominant in age, weight, leg length, and walking experience. The ratio of pressure under the heel was higher, and the periods of heel loading lasted longer than in adults [8,62,63,64,65]. Therefore, the load was concentrated on the anterior calcaneus and a narrower heel, suggesting the need for increased calcaneal robusticity during development to mitigate injury. The load was mainly focused on the anterior calcaneus and narrow heels [8], illustrating the distribution characteristics of the foot loading of toddlers.

Meanwhile, Samson et al. compared different age groups of the ground reaction force, ankle joint, and the metatarsophalangeal joint [31]. As observed in the research of Hallemans et al. [17] and Sutherland [12], the second peak was almost non-existent in vertical force at pre-swing [31] and increased with age [31,69,70]. With increasing walking experience, ground reaction force patterns evolved towards a double-peak (impact and active peak) [17]. Toddlers’ data about the maxima of resistance and propulsion configurations were statistically smaller than adults [31], which indicated that toddlers had an alternative strategy of mainly ankle stabilization and hip propulsion [71]. Biomechanical maturation of joint dynamics occurred approximately age four for the ankle [26].

A large number of studies have shown that in addition to shock absorption as a cushion, the arch also played a vital role in the dispersion and transmission of force in the development process of heel-toe contact mode [4,27,72,73,74,75]. A study found through force transfer algorithm [27] that the pressure and contact area in the middle forefoot increased with toddler development. Strong forefoot support was also found [27,73] and showed the performance of the transverse forefoot arch. Moreover, it was proved that the transverse arch was formed in the early stage (2 years old) by the transfer rule of the load on the forefoot between 2 to 5 years old toddlers. The maximum medial/lateral force decreased with age at early stance, which significantly differed from adults [31]. It was probably due to a decrease in stride width [17]. The windlass mechanism was mature after three years old, causing the load transfer from the middle forefoot to the medial and lateral forefoot. Relevant research proved that the foot can support or transfer loads in the anterior-posterior and media-lateral directions before 6 years old [27]. Thus, the transverse and longitudinal arch appeared in early toddlerhood. The arch promoted force transfer and played an aspirator’s role in the windlass mechanism during walking.

#### 4.3.2. Plantar Pressures of the Toddler Gait

The middle foot area was the main loading area for toddlers to bear body mass [74]. Dulai et al. reported that the impulse in the middle foot area was the highest in the toddler group (2–3 years old) and through the medial forefoot was correspondingly lower [28]. From the perspective of toddler foot morphology and anatomical nature at the onset of independent walking, several studies indicated that the midfoot was full of the fat pad, which was a structure that can effectively relieve the pressure increase caused by weight gain before foot arch maturity [72,75,76,77,78]. Although the arch of toddlers was in the process of continuous development, the middle foot of toddler-aged two has been the transition area connecting the fore and hindfoot [27]. The mid-foot impulse decreased gradually as age advances [27,28], corresponding to the clinical observation of flat feet in toddlers [79].

A longitudinal study on the gait symmetry in typically developing toddlers existed, reporting that the total foot contact area presented symmetry [29]. However, spatiotemporal gait parameters had a certain degree of asymmetry [29,80]. One of the leading causes of foot deformity was caused by asymmetric contact area. In typically developing toddlers aged up to three years old, foot loading patterns might show asymmetric characteristics and become symmetrical with the increase of age and walking experience [29,81]. Joint dynamics were influenced by age during the early childhood [26]. The metatarsophalangeal and ankle joints showed that the maximum eversion moment decreased with age. It was also probable that favoring stability in toddlers [31]. The inverted pendulum mechanism started to mature after three months of walking experience. To minimize energy expenditure, toddlers (at least partially) may use the inverted pendulum mechanism of the energy exchange [32,33], which may not be perfect because of slow walking speed, tossing gait, and smaller kinetic energy fluctuations than potential energy fluctuations. The percentage of mechanical energy recovery increased with walking experience and decreased the gradual variability of kinematic and kinetic parameters [33]. Since each toddler’s speed at which gait matures would be different. Thus, a longitudinal study may be more appropriate to investigate subtle changes and growth [32]. Hallemans et al. [25] reported the kinetics feature of immature gait in toddlers. The dominance of the hip and knee extending moments throughout stance, together with a sustained power production observed around these joints. These findings were supported by the previous reporting [54,82]. Another one was the reduced complexity of the joints (hip, knee, ankle) moment profiles in toddlers, likely caused by immature walking control. The relative rounder shafts of toddlers may be viewed as an early functional adaptation to the unusual demands of the “waddling” locomotion [30].

## 5. Limitations and Future Research

Several other limitations should be considered. The selected kinetic data were limited to the foot and ankle sections. The rotational profile of the lower limbs was not documented. The potential impact of knee joint and hip joint during walking on foot loading symmetry was not included. The laboratory environment could not represent real-world toddler activity. Pressure data collected by walking in a straight line had limitations [35], which reduced gait variability, therefore masking the differences between developmental stages of natural gait [22]. The present results have a particular value, primarily as normative foot loading data, and provide information on the development of foot loading symmetry four years after independent walking. Future studies concerning the typically developing toddler gait may need attention to address a few issues. A particular design may be conducted to analyze empirically before practical application. Research shall systematically recruit subjects with larger samples for a longitudinal study. Anthropometrically and anatomically matched musculoskeletal models may be developed to further decipher the neuro-musculoskeletal biomechanics in the toddler gait [83]. Current techniques, such as the wearables [84] and advanced statistical analysis [85], may also be employed to reveal gait biomechanics. Additionally, the influence of various external environmental interventions on the development of toddler gait also has research value, for example, clothing, footwear, weight-bearing, visual environment interference.

## 6. Conclusions

The study reviewed the biomechanical characteristics of toddler gait at different age stages to independent walking, covering the spatiotemporal parameters, kinematics, and kinetics. The remarkable gait characteristics and typical development nature were reported, indicating that the gait patterns of junior independent walkers differed from senior and experienced cohorts. Gait patterns were associated with the maturation of the neuro and musculoskeletal systems. Changes in gait biomechanics were age and walking experience-dependent. The longitudinal arch played a vital role in the dispersion and transmission of force in developing heel-toe contact. Therefore, it is necessary for pediatric clinicians to understand the characteristics and stages of normal or abnormal development. Knowledge may provide practical implications and healthy references for the diagnosis of gait disorders.

## Figures and Tables

**Table 1:** Literature relating to spatiotemporal parameters of toddler gait.

		Lee et al. [15]		Bisi et al. [16]		Hallemans et al. [17]		Hallemans et al. [18]		Van Dam et al. [19]	
Study site		America		Italy		Belgium		Belgium		Belgium	
Participants		97		20		10		84		100	
Age		10.75 to 19.99 months		10 to 16 months		12.6 ± 1.64 to 17.2 ± 1.64 months		1–10 years (10–36 months included)		15–36 months	
Design		Cross-sectional		Longitudinal		Longitudinal		Cross-sectional		Cross-sectional	
Measurement frequency and timing		Once: 67 infants. 2 or 3 times: 30 infants.		5 times: 0, 1, 2, 3, 6 months after walking onset.		Every 6 months for 9 measurements scheduled.		Once: tested age ranges from 1 to 10 years.		Once: tested age ranges from 15 to 36 months.	
Data Collected		1 walking trial per participant with free play task, 6 consecutive steps per participant with straight-path task.		1 walking trial per participant along the corridor spontaneously.		336 trials of all individuals.		Clear foot strikes on the force plates and full marker visibility for at least 2 consecutive strides.		2 walking trials per participant.	
Anatomic sites		Foot.		Trunk and leg.		Full body (focusing on the lower extremities).		Ankle and foot.		Foot.	
Walking speed and Style		Self-selected speed.		Self-selected speed.		Self-selected speed.		Self-selected speed.		Self-selected speed.	
Footwear/Attire		Not reported		Not reported		Not reported		Bare foot		Soft leather shoes	
Instruments		Pressure-sensitive mat (4 sensors/in2). Three video cameras.		Tri-axial wireless inertial sensors. Video camera.		Instrumented walkway surrounded, infrared cameras (Vicon Motion Systems), force platforms (AMTI).		Optometric movement registration system. Force plate.		Strip of paper of 5 m long and 0.84 m wide. Leather shoes with stamps. Ink.	
Variables		Speed, step length, and step width.		Stride time, swing time, stance time, cadence, acceleration, regularity.		Vertical ground reaction forces, cadence, walking speed, double support time, stride length and step width, joint kinematics and kinetics.		Spatial margin of stability, walking speed, stride length, step width, swing.		Step-time parameters: step length, step width, cadence and walking speed.	
	**Guffey et al. [20]**		**Looper et al. [21]**		**Marencakova et al. [22]**		**Adolph et al. [23]**		**Hamme et al. [24]**		**Hallemans et al. [25]**
Study site	America		America		Czech		America		France		Belgium
Participants	84		8		20		151		106		10
Age	2–4.9 years		6–11 months		7–13 months		11.8–19.3 months		1 to 7 years (10–36 months included)		13.5 to 18.5 months
Design	Cross-sectional		Longitudinal		Cross-sectional		Cross-sectional		Cross-sectional		Cross-sectional
Measurement frequency and timing	Once: 6 age groups split every 6 months.		5 times: 1, 2, 3, 4, 5 months after walking onset.		Once: analyzed age ranges from 7 to 13 months.		Once: tested age ranges from 11.8 to 19.3 months.		several times		Once: tested age ranges from 13.5 to 18.5 months.
Data Collected	3 walking trials per participant.		4 trials of each individual.		1 walking bout consisting of a minimum of 5 complete gait cycles.		1 trials of each individual with free play task and straight-path task.		1 to 6 gait trials per gait analysis.		3–5 trials of each individual.
Anatomic sites	Foot.		Foot.		Leg.		Foot.		Full body (focusing on the lower extremities).		Full body (focusing on the lower extremities).
Walking speed and Style	Self-selected speed.		Self-selected speed.		Self-selected speed.		Self-selected speed.		Self-selected speed.		Self-selected speed.
Footwear/Attire	Bare foot		Not reported		Not reported		Not reported		Not reported		Not reported
Instruments	GAITRite system.		GAITRite system.		Free accessible tool Tracker.		Video camera. GAITRite system.		Instrumented walkway surrounded, infrared cameras (Vicon Motion Systems), force platforms (AMTI).		Instrumented walkway surrounded, infrared cameras (Vicon Motion Systems), force platforms (AMTI).
Variables	Step and stride length, velocity, cadence, step time, cycle time, stance time, swing time, single support time and double support time.		Velocity, cadence, step length, step length and cadence normalized by leg length and single support.		Falls frequency, stops frequency, cadence, time of stance phase, swing phase and double support phase.		Step length, step width, time walking, steps/hour, distance/hour.		Vertical ground reaction forces, angle, moment, power.		Vertical ground reaction forces, cadence, stride time, single support time, double support time, stride length and step width.

**Table 2:** Literature relating to kinematic and kinetic parameters of toddler gait.

	Zeininger et al. [8]	Hallemans et al. [17]	Hallemans et al. [25]	Samson et al. [26]	Hu et al. [27]	Dulai et al. [28]
Study site	America	Belgium	Belgium	France	China	Canada
Participants	18	10	10	75	319	102
Age	11.5 to 43.1 months	12.6 ± 1.64 to 17.2 ± 1.64 months	13.5 to 18.5 months	1 to 6 years (10–36 months included)	2–6 years (10–36 months included)	2–17 years (10–36 months included)
Design	Cross-sectional	Longitudinal	Cross-sectional	Longitudinal	Cross-sectional	Cross-sectional
Measurement frequency and timing	Once: tested age ranges from 11.5 to 43.1 months.	Every 6 months for 9 measurements scheduled.	Once: tested age ranges from 13.5 to 18.5 months.	4 times, 2 times and 1 time per year after 1, 2 and more than 3 of independent walking.	Once: tested age ranges from 2 to 6 years.	Once: 5 age groups (2–3, 4–6, 7–10, 11–14, 15–17 years)
Data Collected	4 trials of each individual.	336 trials of all individuals.	3–5 trials of each individual.	6 trials of each foot on force plate.	3 walks for each foot.	3 trials of each individual.
Anatomic sites	Total foot.	Full body (focusing on the lower extremities).	Full body (focusing on the lower extremities).	Ankle, knee and hip joints	Hallux, toes, metatarsal heads, mid-foot, medial and lateral heel.	Hallux, heel, lesser toes, medial and lateral forefoot, midfoot.
Walking speed and Style	Self-selected speed.	Self-selected speed.	Self-selected speed.	Self-selected speed.	Self-selected speed.	Not reported.
Footwear/Attire	Bare feet	Not reported	Not reported	Not reported	Not reported	Bare feet
Instruments	Vicon MX motion analysis system synchronized with Bertec force plates.	Instrumented walkway surrounded, infrared cameras (Vicon Motion Systems), force platforms (AMTI).	Instrumented walkway surrounded, infrared cameras (Vicon Motion Systems), force platforms (AMTI).	Motion Analysiss system with Eagles cameras, force platform, Footscans plantar pressure platform.	RSscan (4 sensors cm^2^).	emed-x platform (4 sensors cm^2^).
Dynamic variables	Location of the center of pressure relative to the calcaneus, the orientation and magnitude of ground reaction forces during foot contact.	Vertical ground reaction forces, cadence, walking speed, double support time, stride length and step width, joint kinematics and kinetics.	Vertical ground reaction forces, cadence, stride time, single support time, double support time, stride length and step width.	Moment, speed.	Relative force-time integral (FTIrel) (%).	Foot impulse, regional percent impulses, impulse ratios.
	**Bosch et al. [29]**	**Cowgill et al. [30]**	**Samson et al. [31]**	**Hallemans et al. [32]**	**Ivanenko et al. [33]**	**Ivanenko et al. [34]**
Study site	Germany	America	France	Belgium	Saint Lucia	Saint Lucia
Participants	62	10	42	9	26	7
Age	15.1 ± 2.4 to 63.2 ± 2.4 months	1 to 3.9 years	1 to 6 years (10–36 months included)	12 to 18 months	11 to 153 months (10–36 months included)	12 to 15 months
Design	Longitudinal	Cross-sectional	Cross-sectional	Cross-sectional	Cross-sectional	Cross-sectional
Measurement frequency and timing	Every 6 months for 9 measurements scheduled.	Once: tested age ranges from 1 to 3.9 years.	Once: tested age ranges from 1 to 6 years.	Once: tested age ranges from 12 to 18 months.	Once: tested age ranges from 13.5 to 18.5 months.	Once: tested age ranges from 12 to 15 months.
Data Collected	5 trials of each individual.	1 trail of 1 foot on force plate and speed changed less than 20%.	1 trials of each foot on force plate.	5 trials of each individual.	10 trials of each individual.	10 trials of each individual.
Anatomic sites	Total foot.	Total foot, greater trochanter, lateral femoral condyle and lateral malleolus.	Total foot, metatarsophalangeal and ankle joint.	Full body.	Trunk, pelvis, thigh, shank, and foot.	Trunk, pelvis, thigh, shank, and foot.
Walking speed and Style	Self-selected speed.	Self-selected speed.	Self-selected speed.	Self-selected speed.	Self-selected speed.	Self-selected speed.
Footwear/Attire	Bare feet	Bare feet	Not reported	Not reported	Not reported	Not reported
Instruments	capacitive pressure distribution platform (4 sensors cm^2^).	infrared cameras (Vicon MX4) with skin marker attachment and embedded force plate (AMTI).	Motion Analysiss system with Eagles cameras, force platform, Footscans plantar pressure platform.	Instrumented walkway surrounded, infrared cameras (Vicon Motion Systems), force platforms (AMTI).	ELITE or VICON motion analysis systems, force platform.	VICON motion analysis systems, force platform.
Dynamic variables	Contact area, peak pressure, force-time integral, relative maximum force, contact time and the force-time integral.	Peak ground reaction forces and ground reaction forces impulse.	Vertical ground reaction forces, moment.	Vertical ground reaction forces, speed, moment.	Vertical ground reaction forces, speed, moment.	Vertical ground reaction forces, speed, step length and width.

## Data Availability

The data concerning all literature included may be available upon request from corresponding authors.
